# Proceedings of an expert workshop on community agreement for gene drive research in Africa - Co-organised by KEMRI, PAMCA and Target Malaria

**DOI:** 10.12688/gatesopenres.13221.2

**Published:** 2021-03-24

**Authors:** Delphine Thizy, Lea Pare Toe, Charles Mbogo, Damaris Matoke-Muhia, Vincent Pius Alibu, S. Kathleen Barnhill-Dilling, Tracey Chantler, Gershom Chongwe, Jason Delborne, Lydia Kapiriri, Esther Nassonko Kavuma, Sethlomo Koloi-Keaikitse, Ana Kormos, Katherine Littler, Dickson Lwetoijera, Roberta Vargas de Moraes, Noni Mumba, Lilian Mutengu, Sylvia Mwichuli, Silvia Elizabeth Nabukenya, Janet Nakigudde, Paul Ndebele, Carolyne Ngara, Eric Ochomo, Simon Odiwuor Ondiek, Stephany Rivera, Aaron J. Roberts, Benjamin Robinson, Rodrick Sambakunsi, Abha Saxena, Naima Sykes, Brian B. Tarimo, Nicki Tiffin, Karen H. Tountas

**Affiliations:** 1 Imperial College London, London, UK; 2 Institut de Recherche en Science de la Santé, Ouagadougou, Burkina Faso; 3 Kenyan Institute of Medical Research, Kilifi, Kenya; 4 Pan African Mosquito Control Association, Nairobi, Kenya; 5 Kenyan Institute of Medical Research, Nairobi, Kenya; 6 Makerere University, Kampala, Uganda; 7 North Carolina State University, Raleigh, USA; 8 London School of Hygiene & Tropical Medicine, London, UK; 9 Tropical Diseases Research Centre, Ndola, Ndola, Zambia; 10 Department of Health, Ageing and Society, McMaster University, Hamilton, Canada; 11 Jenak Investments Limited, Kampala, Uganda; 12 faculty of education, University of Botswana, Gaborone, Botswana; 13 University of California Irvine Malaria Initiative, Irvine, USA; 14 Global Health Ethics Unit, World Health Organization, Geneva, Switzerland; 15 Ifakara Health Institute, Dar-Es-Salaam, Tanzania; 16 Institute on Ethics and Policy for Innovation, Faculty of Humanities, McMaster University, Hamilton, Canada; 17 African Academy of Sciences, Nairobi, Kenya; 18 International Center for Evaluation and Development, nairobi, Kenya; 19 Infectious Diseases Institute, Kampala, Uganda; 20 College of Health Sciences, Makerere University, Kampala, Uganda; 21 Milken Institute School of Public Health, George Washington University, Washington DC, USA; 22 Wellcome Trust,UK, London, UK; 23 Kenyan Institute of Medical Research, Kisumu, Kenya; 24 Emerging Ag., Calgary, Alberta, Canada; 25 Malawi Liverpool Wellcome Trust Clinical Research Program, Blantyre, Malawi; 26 The INCLEN Trust International, Delhi, India; 27 Institut Ethique Histoire Humanités, University of Geneva, Geneva, Switzerland; 28 Vector Immunity and Transmission Biology Unit, Department of Environmental Health and Ecological Sciences,, ifakara Health Institute, Bagamoyo, Tanzania; 29 Division of Computational Biology, and Wellcome Centre for Infectious Disease Research in Africa, University of Cape Town, Cape Town, South Africa; 30 Foundation for the National Institutes of Health, Bethesda, USA

**Keywords:** Consent, gene drive, stakeholder engagement, agreement, community acceptance, genetically modified mosquitoes

## Abstract

Gene drive research is progressing towards future field evaluation of modified mosquitoes for malaria control in sub-Saharan Africa. While many literature sources and guidance point to the inadequacy of individual informed consent for any genetically modified mosquito release, including gene drive ones, (outside of epidemiological studies that might require blood samples) and at the need for a community-level decision, researchers often find themselves with no specific guidance on how that decision should be made, expressed and by whom. Target Malaria, the Kenya Medical Research Institute and the Pan African Mosquito Control Association co-organised a workshop with researchers and practitioners on this topic to question the model proposed by Target Malaria in its research so far that involved the release of genetically modified sterile male mosquitoes and how this could be adapted to future studies involving gene drive mosquito releases for them to offer reflections about potential best practices. This paper shares the outcomes of that workshop and highlights the remaining topics for discussion before a comprehensive model can be designed.

## Disclaimer

The views expressed in this article are those of the authors. Publication in Gates Open Research does not imply endorsement by the Gates Foundation.

## Background

Recent scientific advances in the field of gene drive research have increased the likelihood that gene drive-modified mosquitoes to reduce the burden of malaria will be proposed for field evaluation in the near-to-medium-term future. This technology is considered a potentially transformative tool for malaria elimination ( Feachem *et al.*, 2019; World Health Organization, 2020a), due to some of its characteristics including the absence of behaviour change associated with it, its egalitarian nature and its ability to cover hard-to-reach locations. As several of the leading research teams in this field are currently working in Africa, where the burden of malaria is most significant ( World Health Organization, 2020c), interest on that continent in the research is particularly strong ( African Union, 2018).

Stakeholder engagement is an essential part of responsible gene drive research ( James *et al.*, 2018; National Academies of Sciences Engineering and Medicine, 2016; Resnik, 2018) and increasingly for all public health research and in particular that on vector-borne diseases. However, because genetic approaches and gene drive specifically are reasonably new area-wide technologies in the field of vector control, many theoretical and practical components of ethical and effective engagement strategies remain subject to debate. Those discussions are not specific to gene drive, they apply to other genetic approaches and to other area-wide vector control measures, but gene drive technologies seem to have put this question under a new spotlight ( World Health Organization, 2020b). While area-wide control technologies can offer the benefit of providing vector control for all inhabitants of a specific area without individual or group biases related to economic status, education or social position, it may not be possible for individuals within a given community to opt-out of field evaluations. The World Health Organisation (WHO) *Guidance framework for testing genetically modified mosquitoes* clearly establishes that individual consent is not appropriate for the open release of genetically-modified mosquitoes unless there is a collection of samples or data from human participants to track epidemiological results, in which cases those collections require individual consent ( WHO/TDR and FNIH, 2014). Instead, the WHO guidance calls for "community authorisation" prior to the release of genetically modified mosquitoes. However, there is currently no established consensus on definitions or guidelines related to community authorisation in gene drive research, or on how research teams should approach these issues in preparation for field evaluations.

Given their shared interest in the issue of malaria control and shared expertise in community and stakeholder engagement, Target Malaria, Kenya Medical Research Institute (KEMRI), and Pan African Mosquito Control Association (PAMCA) partnered to convene experts and researchers to discuss the question of how to approach community acceptance for field evaluations of gene drive mosquitoes.

As one of the leading research projects in the field of gene drive mosquitoes for malaria control, and to support its commitment to conduct its research according to the best scientific and ethical standards and practices, Target Malaria has actively undertaken outreach to other research groups – such as those working on *Wolbachia* approaches for diseases transmitted by *Aedes* mosquitoes such as the World Mosquito Programme or the National Environment Agency of Singapore or other groups working on vector control in Africa such as KEMRI, Ifakara Health Institute – and to additional experts on stakeholder engagement, deliberative dialogue and bioethics to help co-develop and establish best practices for stakeholder engagement. It has already collaborated with partners on the broader questions of best practices for stakeholder engagement for the research and development of genetically modified mosquitoes (incl. releases), resulting in the publication of several papers on the subject ( Barry *et al.*, 2020; Hartley *et al.*, 2019; Thizy *et al.*, 2019) documenting its approach to co-development, the work with local communities, and proposing guidance for technology developers in gene drive research and other fields.

Kenya Medical Research Institute: KEMRI is a State Corporation responsible for carrying out research for human health in Kenya. KEMRI's mandate is to oversee the research activities with a view to providing advice and direction for national development. KEMRI provides leadership in health research & development, shaping the health research agenda, setting norms and standards, articulating evidence-based policy options and monitoring and assessing health trends. In addition, KEMRI deals with trans-boundary threats and disease outbreaks. The safe and ethical development of new technologies relevant to public health, therefore, falls under its remit. KEMRI has developed a critical mass of scientists, technical and administrative support staff to rank as one of the leading centres of excellence in health research and development in the African region and beyond.

Pan African Mosquito Control Association: PAMCA is an African professional body that brings together actors in the field of vectors and vector-borne disease control. PAMCA exists to provide a platform for capacity building, knowledge sharing and collaboration for concerted vector control initiatives in the African continent. The mission of PAMCA is to provide a platform for Africa-based scientists, public health professionals and other stakeholders to drive efforts towards control and elimination of mosquito-borne diseases. PAMCA has an increasing number of well-trained entomologists distributed across the continent who help to address local problems with locally adapted solutions and through partnerships between national mosquito control programs, research institutions, product development companies and funders involved in mosquito control on the continent. Since 2017, PAMCA has partnered with Target Malaria to train more than 60 early-career scientists on gene drive technology and its application for malaria control.

The objective of this expert workshop on community agreement for gene drive research in Africa was to provide direction and recommendations to Target Malaria and beyond the project offer some reflections for other projects, specifically on the question of community acceptance and consent for possible future field evaluations of gene drive-modified mosquitoes.

While the purposes of stakeholder engagement are multiple and go well beyond seeking acceptance of the technology and field trials, researchers must grapple with what constitutes meaningful acceptance as this is a requirement for their research to be able to proceed. The workshop aimed to clarify what is meant by agreement, consent and acceptance, what practices, requirements and standards could be drawn upon by Target Malaria to ensure decisions are informed and meaningful, and what are the responsibilities and roles of researchers and other actors in this process.

## Workshop description

The workshop was intended both to inform Target Malaria's work and to build a community of practice that the project can refer to in future as it seeks guidance. Participants were drawn from the professional networks of Target Malaria, KEMRI, and PAMCA based on relevant expertise, experience, and interest, while ensuring regional and gender balance. After an initial briefing that provided overviews on gene drive technology and Target Malaria's community consent model and experience to-date, structured discussions took place over a 5-week period, hosted on the MESH Community Engagement Network (MESH), a networking and dialogue space for researchers focused on community engagement and global health. Each participant is included in the author list or in the acknowledgements.

MESH-hosted discussions were structured according to three thematic clusters: 1) representation and legitimacy, 2) accountability and 3) operational considerations. These were followed by a fourth discussion during which participants sought to translate the concepts and issues raised into "best practices". For each theme, a selection of guiding questions was provided. These were intended to stimulate conversation, but not confine the dialogue; participants were, therefore, free to address the questions or ignore them according to their perceived relevance. The list of guiding questions can be found in Box 1. Online virtual working meetings were held concurrently with each MESH-hosted discussion to provide an interactive venue for participants to address these topics openly, and to encourage the participation of those less able or willing to contribute to writing due to time, language, or other constraints. The combination of MESH-hosted discussions and virtual working meetings allowed the greatest possible range of participants to contribute actively. Virtual working meetings were hosted on Zoom and accompanied by an interactive visual hosted on Mural that all participants had access to. In between virtual working meetings, participants were invited to make further comments or changes on the Mural visuals to reflect any additional points they wanted to make. All findings were drawn from the MESH-hosted discussions, the interactive MURAL visuals, and notes taken during the virtual working meetings.

**Box 1. Guiding questions**

MESH-hosted discussions were structured according to three thematic clusters (listed below), followed by a fourth discussion during which participants sought to translate the concepts and issues raised into "best practices". For each theme, a selection of guiding questions was provided. These were intended to stimulate conversation, but not confine the dialogue; participants were, therefore, free to address the questions or ignore them according to their perceived relevance. These questions are included here for reference. They were elaborated by Target Malaria and the Emerging Ag facilitating team on the basis of challenges faced by the project in the past or concerns raised by stakeholders or media in the dialogue on this research.

**Representation and legitimacy**

How are communities defined and delimited? Who is considered a member of a specific community and who isn't? Who makes those judgements?

Are there criteria to determine whether "representatives" or "representative groups" are truly representative of the interests and composition of a community? What can or should Target Malaria do in cases where this is found not to be the case?

How can tensions between culturally-appropriate representation and decision-making models and representation of vulnerable groups be addressed or managed?

How should the project engage with members of the community who may be opposed to the research? Mainly if their opinions are determined not to be representative of those of the community?

How should the project engage with stakeholders who may not be part of the community, and so may not be obliged to consent to research, but who nonetheless feel they have a stake? For example, non-local NGOs opposed to GM research?

**Accountability**

What baseline obligations do project research teams have towards stakeholders when seeking community consent? How will these obligations change over the course of a project's lifetime?

How can Target Malaria assess whether they are currently falling short of any of these obligations? What are the most appropriate mechanisms for doing so?

How can Target Malaria ensure that the complaint processes and grievance mechanisms established are effective and inclusive?

Under what circumstances is it beneficial to involve actors external to the project (and who may not be directly affected stakeholders) in the consent process? Who might those actors be? Should they be involved during the information sharing process in order to address perceptions of bias (i.e. concerns that those developing the technology are the only ones providing information about it?

What role should external auditors/certifiers play in project activities related to the consent-seeking process?

**Operational considerations**

How can Target Malaria's current or planned approaches to seeking community consent be adequately scaled to different sized releases?

What adaptations or changes may need to be made, and how can these be done without undermining the legitimacy of the process and decisions?

Once consent has been obtained, are there further roles for the community to play in Target Malaria's research? If so, what should they be?

**Best practices**

Drawing on previous discussions, are there best practices for seeking community consent that can be identified that are of particular relevance to Target Malaria?

Do any of these best practices need to be prioritised over others?

Are there issues or considerations related to community consent that still require further work or reflection on behalf of Target Malaria to resolve?

Are there other projects or processes, or other relevant organisations, that have drawn-up benchmarks or frameworks that could inform Target Malaria's work?

What roles and responsibilities can ethics committees be expected to take on, and how can this be reflected in best practices? What about national authorities?

## Workshop findings

Findings are presented according to the key themes that arose in the course of discussions.

**Appropriate conceptual terminology:** The term "consent" has particular connotations in the realms of ethics and clinical trials that may not be appropriate to transpose from the field of health research with human participants to field experiments involving gene drive-modified mosquitoes, as this understanding of consent is rooted in notions of individual rights and autonomy. Free prior and informed consent" (FPIC) is another conception of consent related to collective approval that is prevalent in (among others) the realms of conservation and the rights of indigenous peoples and affected communities ( George *et al.*, 2019). This connotation of consent is also less appropriate to the work of Target Malaria, as the project is not currently active in areas where there are recognised indigenous peoples and the conceptual definitions that underpin its work should be applicable to all relevant stakeholders not merely those directly affected by its efforts. In light of this, participants found that "community agreement" was the least ambiguous and most fitting conceptual terminology for Target Malaria to seek from relevant communities before engaging in research that may impact them. Participants felt that "community agreement" implied a two-way discussion between relevant stakeholders and the project in which stakeholders have a more significant influence in helping to define the terms and expected outcomes of the engagement process, in contrast with other terminologies (such as "community acceptance)". When attempting to determine what constituted "community agreement", participants settled broadly on "permission provided for research activities to proceed according to conditions negotiated during the engagement process, with all relevant stakeholders having provided informed approval".

**Identifying the relevant stakeholders and communities with which to engage.** One of the most challenging topics that participants discussed was determining the relevant communities whose agreement must be sought for each phase and stage of research. The self-sustaining characteristics of some gene drive technologies (such as the ones considered by Target Malaria) make this particularly complex, as the members and delineations of relevant stakeholder communities may change over time as the gene drive intervention persists in the environment and/or spreads spatiotemporally. It was noted therefore that while the geographic location of stakeholders is essential, the stakeholders' relationship to the ecologies and landscapes in which the species targeted by a specific gene drive intervention is embedded also may need to be considered. A distinction was drawn between relevant communities – those with tangible and immediate interests in Target Malaria's work and/or who bear substantial and immediate risks related to it – and the general public. This is in line with definitions proposed by Lavery *et al.* ( Lavery *et al.*, 2010) and the National Academies of Sciences, Engineering, and Medicine ( National Academies of Sciences Engineering and Medicine, 2016). Although researchers may not be obliged to seek the agreement of the general public for research to proceed, Target Malaria may wish to engage in less intensive, but parallel and complementary forms of outreach, such as disseminating information about the research through mass media, keeping an open dialogue with this public and consulting with regional and national authorities.

**Figure 1.**
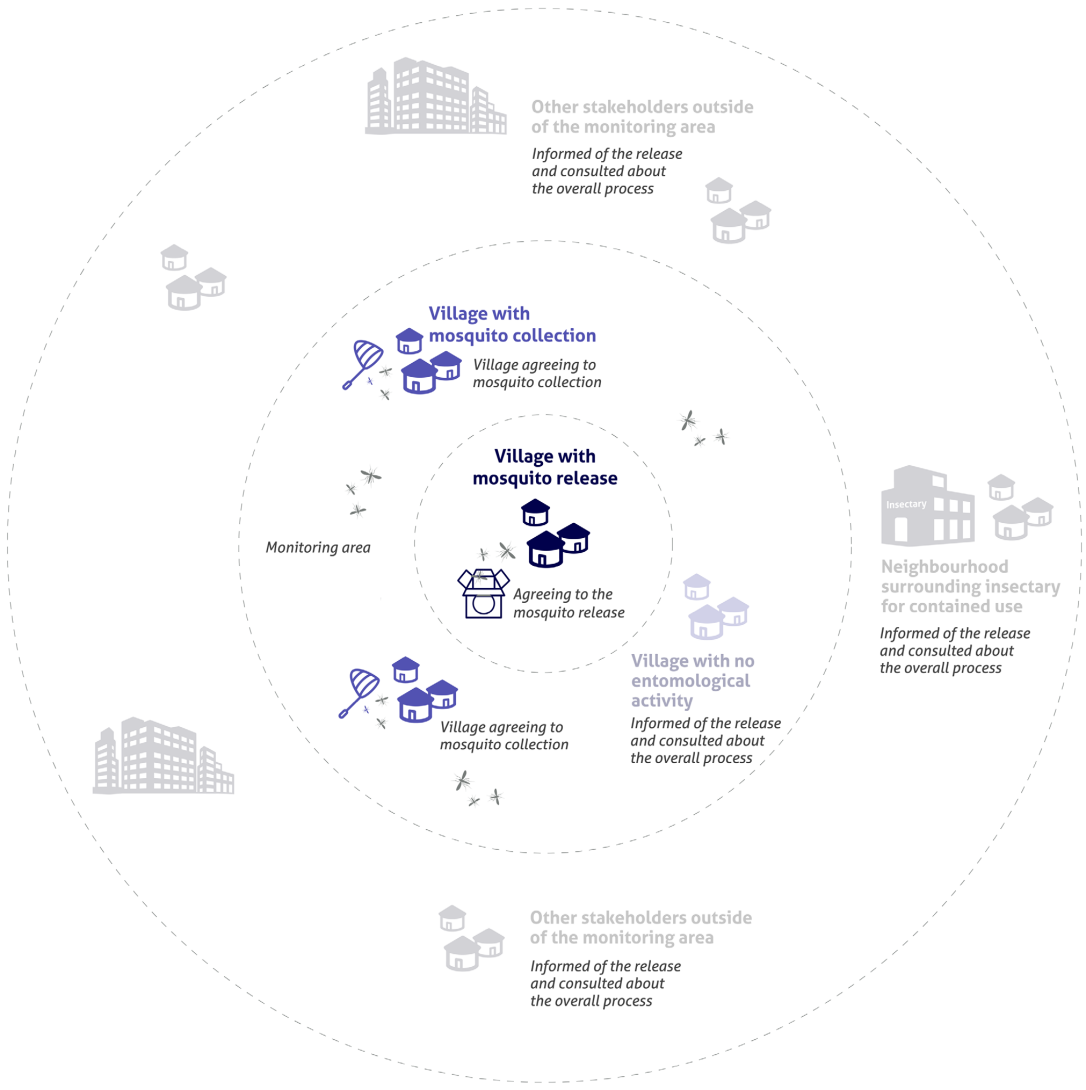
Areas of differing stakeholder engagement.

With regards to relevant communities, further consideration was given to the differences that could exist between residents of areas where research activities take place, including potential release sites for field evaluations, and residents of areas where research activities do not take place directly but which would be included in the monitoring area after releases of gene drive-modified mosquitoes (see Figure 1). Participants considered whether the concept of "bystanders", as outlined by Walen ( Walen, 2020) would be applicable to the work of Target Malaria. This definition would draw a distinction between those residing in an area potentially affected by a gene drive field trial (bystanders) and those who interact directly with, or host, research activities. The requirements for agreement from the former may be lesser. Some participants contended that this definition would be irrelevant in the case of trial gene drive releases, as there are no specific human beings who are directly experiencing the intervention so that all stakeholders would be bystanders. While Walen's definition may hence not be directly useful, participants agreed that the challenge posed by gene drive-modified mosquitoes which, by design, are intended to spread geographically and establish themselves beyond the release points, requires careful consideration of how to determine the scope of the relevant communities. While all communities in the area impacted by the research should be engaged and informed, it was suggested that the model for agreement and representation could be different for communities in release sites and those in the monitoring areas. Researchers could work in a more devolved or mediated model with the monitoring area communities, for example, through local authorities to share information and gather feedback from those communities on research activities. These authorities could be key to brokering community agreement.

Once relevant communities have been identified at the theoretical level, there remains the often-challenging task of translating theory into practice. Participants agreed that the stakeholders from the relevant communities, whether the project activity that concerns them is direct research, monitoring, or something else, must be involved in decisions that delineate the bounds of their communities and in designating their own representatives. In doing this, project teams must strike a balance between consulting those recognised as having authority delegated to them by the community (elders, headmen, elected representatives etc.) and taking into account cultural or communal biases that may lead to the neglect of concerns expressed by some stakeholder groups (for example women, ethnic minorities, those with disabilities). Relevant authorities who could play an advisory or decision-making role may include national regulatory agencies and ethics committees, formal and informal community leaders, representatives of marginalised groups and others.

Formative research, including anthropological research, to understand demographic and institutional profiles, social and cultural mores, relationships of power etc. should help inform the community engagement process. Engagement with authorities and other community representatives should not preclude broader forms of engagement with the community as a whole (radio broadcasts, town halls meetings, didactic theatre performances, etc.), which can help ensure that information is sufficiently disseminated and decisions regarding any community agreement are adequately informed and can help prevent the spread of misinformation

Remaining questions for further discussion:

- How is a community defined? It was determined that further information and research is needed before the workshop participants would be able to articulate a broad consensus. The concept of community has been discussed for genetically modified mosquitoes' field release, including that of gene drives, and some definitions have been suggested ( James *et al.*, 2018; National Academies of Sciences Engineering and Medicine, 2016; Resnik, 2018). However, because Target Malaria's research on gene drive-modified mosquitoes is still in the laboratory stages, the rate of geographical and temporal dispersal of such mosquitoes is unknown at this point. The development of models for the spatiotemporal spread of future gene drive-modified mosquitoes for field trials will depend on a number of variables, including release protocol, the characteristics of the gene drive-containing DNA construct, and ecological factors. All of which may evolve over time as the research project progresses. This will have profound implications for how relevant communities that need to be engaged for community agreements are defined. Indeed, if dispersal is great enough then the relevant communities may not share mutual interests, culture, or mechanisms of governance, straining the project's concept of "community" and the ability of researchers to utilise a similar or generic approach for engagement with different groups. It was noted that this might be of more significant concern for the possible use of any developed technology, rather than during Target Malaria's near-and-medium-term research activities, involving the releases of non-gene drive genetically modified mosquitoes that are self-limiting by nature and thus do not affect such a large geographical area.

**Engaging with stakeholders who may not wish for the research to take place and taking their opposing views into consideration.** As mentioned earlier, one of the signal challenges of testing area-wide vector control methods (such as those being developed by Target Malaria), is the inability of individuals within an agreeing community to opt-out, due to the nature of the technology. During the process of seeking community agreement, venues and channels must be available for those opposed to the research going forward to voice their opinions to researchers and other stakeholders. This is common in any kind of community engagement effort ( International Finance Corporation, 2007; Wood, 2011). The community agreement brokering process, including the avenues for expressing disagreement, must be elaborated ahead of the engagement process in consultation with the stakeholders themselves, to ensure that those who may not find themselves in accordance with the outcomes in the community agreement are at least in accordance with the process that produced it. Even after the terms of a community agreement are attained, those who disagree with the outcomes can provide valuable insight into the work of the project. A comprehensive engagement process can help build trust and perceptions of procedural justice by demonstrating that their perceptions were actively and seriously considered. 

In addition to this critical process for decision making, itself the product of a method of dialogue between communities and researchers, it was noted that agreement achieved at one point in time should not be considered final and irrevocable across, and for the remainder of, the project. Research like Target Malaria's takes place over years and agreement is sought from communities multiple times for many different activities along the research continuum over an extended period of time, not only at the moment of seeking to carry out field evaluations. Communities will have multiple and renewed opportunities to seek changes to the terms of a community agreement or to end the agreement. Therefore, the community agreement process should be thought of as a dynamic engagement process with multiple decisions and agreements that offer multiple opportunities for different views to be expressed, heard, and negotiated. 

Remaining questions for further discussion:

- What if the geographical scope of a field trial encompasses a large enough number of people that they would be considered multiple communities according to the criteria outlined in "remaining questions for further discussion" above? In this case, if communities were to differ as to the acceptability of the research taking place, a legitimate and culturally conversant mechanism for intra-community deliberation would need to be elaborated. What shape such a tool might take would require further work. This may be a significant issue; however, this is not urgent, as experimental trials on that scale involving gene drive-modified mosquitoes are unlikely to take place in the medium-term future. This would only be relevant for activities which would affect all communities in a field trial site.

**Monitoring mechanisms.** Of equal importance in determining who should be consulted and on what terms, is the question of how the terms of a community agreement can be monitored or verified to hold researchers accountable for fulfilling their responsibilities. Monitoring mechanisms independent of researcher project teams can help minimise perceived or actual conflicts of interest. On the other hand, independent monitoring by external entities may contribute to undermining community trust by creating the perception that their feedback and monitoring methods alone are insufficient to confirm compliance with the terms of the community agreement. In addition, independent monitoring activities may contribute to a perception that researchers are engaged in the form of legal or regulatory "box-ticking" for the benefit of other stakeholder audiences, not for the benefit of the community itself. Therefore, any monitoring mechanisms must be established in active consultation with affected communities and should be part of the agreement negotiated with the community. The need for such independent monitoring will depend heavily upon the context in which the research is taking place, and relevant external authorities may include governmental regulatory authorities, third party NGOs, professional auditors, community advisory boards and others. In some cases, such third parties are well established and have a predefined monitoring role (for example national biosafety authorities), in other cases, they may need to be identified or created (such as community advisory boards, external professional auditors). Some practitioners noted that the lack of an external authority has in the past led to perceptions among stakeholders of a lack of appropriate oversight.

Target Malaria is one of the first projects to work on gene drive approaches for malaria control, and it is likely to be the first of its kind to work through some aspects of stakeholder engagement and community agreement related to this technology. As such, the project's experiences and reflections related to the process of seeking community agreement are likely to be extremely valuable to other gene drive researchers in future. Therefore, participants felt that in addition to its own internal tracking and documentation processes, Target Malaria might find working with an independent evaluator of its stakeholder engagement and community agreement efforts to be valuable.

Remaining questions for further discussion:

- How will the appropriate criteria and benchmarks be selected and by whom to ensure an evaluation process is useful?
- What parameters will ensure that the evaluation will be sufficiently independent? External sources of funding for an assessment may be minimal, whereas having the evaluation funded by the project itself risks calling into question its autonomy.

**Figure 2.**
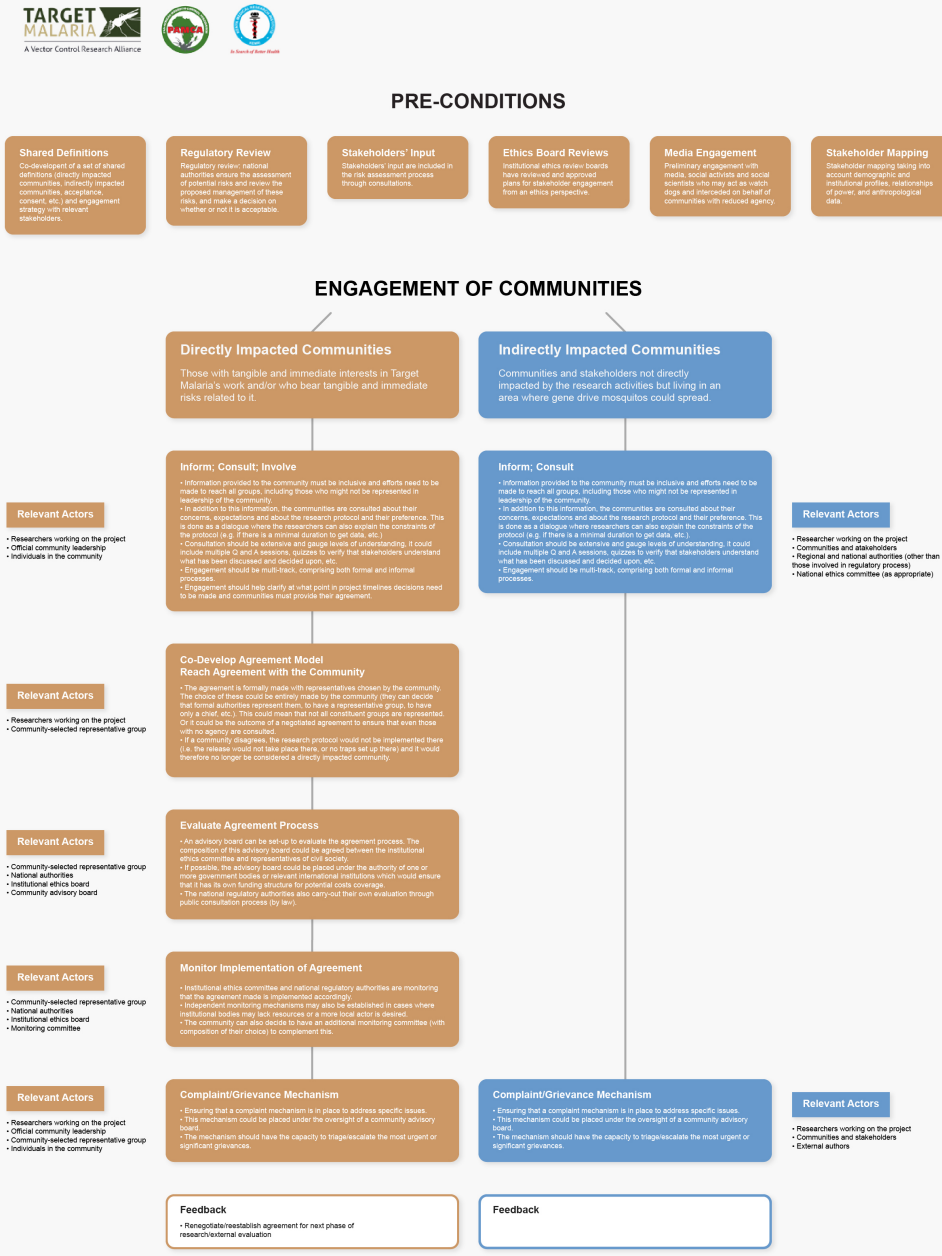
Operational model.

**Operationalising these considerations.** Participants were asked how the workshop findings discussed above could be integrated into a model of Target Malaria's engagement and community agreement seeking processes. A visual model reflecting the outcomes was developed, which can be found in Figure 2. It establishes the pre-conditions necessary for successful community engagement and lays out two separate pathways for engagement depending on whether the community in question will be directly or indirectly affected by research activities while identifying the relevant actors for each stage in the engagement process.

## Conclusion

During this workshop participants helped identify good practices and key considerations that Target Malaria can incorporate into its stakeholder engagement and community agreement-seeking processes as it moves forward with its research, as well as areas in which further thought and greater conceptual clarity are needed. These considerations are likely to be broadly applicable to many different gene drive research projects, as well as research involving field releases of genetically modified insects.

Key workshop considerations are summarised below:

1. Appropriate conceptual terminology: Participants found that "community agreement" – defined here as permission provided for research activities to proceed according to conditions negotiated during the engagement process, with all relevant stakeholders having provided informed approval – was the least ambiguous and most fitting conceptual terminology for Target Malaria to seek from relevant communities.
2. Identifying the relevant stakeholders and communities with which to engage: 'Relevant communities' and the 'general public' were deemed distinct, and, for Target Malaria, relevant communities were further delineated as sites for field evaluations and areas where monitoring may take place, with particular models of stakeholder/community engagement in the two.
3. Engaging with and taking into consideration opposing community stakeholder views: Target Malaria's community agreement process should be thought of as a series of multiple engagements, decisions and agreements that offer opportunities for the full range of views to be expressed, heard, and negotiated.
4. Monitoring mechanisms: Monitoring mechanisms independent of Target Malaria activities can help minimise the perception of or actual conflicts of interest.
5. Operationalising these considerations in practice: See Figure 2.

In addition, participants identified areas in which further thought and greater conceptual clarity are needed.

- How is 'community' defined?
- What if the geographical scope of a field trial encompasses a large enough number of people that they would be considered multiple communities?
- How will the right criteria and benchmarks be selected and by whom to ensure a monitoring evaluation process is useful?
- What parameters will ensure that a monitoring evaluation will be sufficiently independent?

Beyond the specific conclusions of this workshop, it was an opportunity for participants to share their knowledge, perspectives and opinions on this crucial topic of community-agreement seeking for gene drive field evaluation. Several participants volunteered to be consulted again in the future about the model or about specific aspects of it, such as some of the points highlighted for further discussion. For Target Malaria, KEMRI and PAMCA, this was a chance to start creating a community of practice with experts from the African continent and beyond, who could be further consulted on this issue in the future and who through time are building a shared understanding of the subjects at hand. The adaptation of the workshop format from an in-person meeting to a virtual meeting due to the coronavirus disease 2019 (COVID-19) pandemic created challenges for the format but has allowed broader participation from experts who might not have been able to dedicate three consecutive days to travel to an in-person meeting.

## Data availability

### Underlying data

No data are associated with this article.
