# Supplementary material for: Proceedings of an expert workshop on community agreement for gene drive research in Africa - Co-organised by KEMRI, PAMCA and Target Malaria
Source: Gates Open Res. 2021 Mar 24;5:19. Originally published 2021 Jan 29. [Version 2] doi: 10.12688/gatesopenres.13221.2 (PMC8042295; doi:10.12688/gatesopenres.13221.2)
Supplement: Peer review history

# Peer review: Reviewer response for version 2

**DOI:** 10.21956/gatesopenres.14454.r30517

**Published:** 2021-04-12

**Authors:** Sam Weiss Evans

**Affiliations:** 1 Program on Science, Technology, and Society, Harvard University, Cambridge, MA, USA

The authors of this Open Letter outline the work that they have done to build capacity for community engagement that can lead to "community agreement" for the field testing of gene drive mosquitos. 

 As a workshop report, this paper seems adequate, but there are important gaps that this paper does not address which bear mention here in this public peer review. They fall under two basic categories:

1. We should not assume that democratic engagement at the point of field trials could result in meaningful reconfiguration of the technology itself; and 
2. The lack of attention to the opposite of "community agreement" means that there is no clear way for communities to express their desire to not have the research move forward in their locale.

Gene drive work is necessarily political as well as technical, and thus is as much about re-forming the social orderings within a community as well as the technical orderings in it and the environment around it. The authors' attention to the construction of the "community" appropriately captures this dynamic, if not calling it out directly. While attention is given in the letter to ensure that the authority of local forms of government are acknowledged even as they are reconfigured in the process of reaching "community agreement," it is unclear how much attention there is to the authority of those local forms of government's ability to re-form the technologies under consideration.

 The choice of what types of political arrangements are acceptable to the authors is shown at several points, with a strong preference for various forms of democratic input (e.g. stakeholders accepting the process of deliberation even if they do not accept the outcome).

 One of the elements that appears to be missing in this paper is attention to feedback loops between communities and actual research design. There are several points in the paper where the focus is (appropriately) on the feedback loop on the ways that the engagement effort itself is structured, but not on how feedback reaches further back to lab development work. 

 As a community engagement paper specifically around the moment of decision-making on field trials, this may be adequate, but that would assume that earlier feedback loops on research design and lab development also took place. There was no mention in the paper of any such prior engagement leading to feedback and alteration of research designs, leading the reader to assume that this is a technology developed in the West and tested in the global south, an accusation I suspect the authors are keen to counter. The Hartley *et al.* paper cited only notes that "tools to track knowledge flows from publics to scientists are less developed." They note that Target Malaria was, in 2019, setting up an "ecological observatory" in Ghana, but this was still preliminary. The Thizy *et al.* paper was a general statement about the need for early and continuous engagement, but with no specific examples on how Target Malaria was actually doing that. 

 There was also no discussion of what would count as the opposite of "community agreement" - that term is solely reserved for "permission provided to research activities to proceed." There is no option, for example, for there to be "community agreement for research to not proceed." This lack of an alternative decision to a "yes" means that there is no clear method for there to be a definitive "no." 

 In short, there is a strong assumption of the value of community input through means that place the community on an epistemic par with the researchers at a specific point in the research lifecycle for gene drives: the initiation of field trials.

Does the article adequately reference differing views and opinions?

Yes

Are all factual statements correct, and are statements and arguments made adequately supported by citations?

Yes

Is the Open Letter written in accessible language?

Yes

Where applicable, are recommendations and next steps explained clearly for others to follow?

Yes

Is the rationale for the Open Letter provided in sufficient detail?

Yes

Reviewer Expertise:

very early-stage community engagement, science and technology studies, governance anomalies in science and technology

I confirm that I have read this submission and believe that I have an appropriate level of expertise to confirm that it is of an acceptable scientific standard.

# Peer review: Reviewer response for version 2

**DOI:** 10.21956/gatesopenres.14454.r30570

**Published:** 2021-04-08

**Authors:** David B. Resnik

**Affiliations:** 1 National Institute of Environmental Health Sciences, National Institutes of Health (NIH), Bethesda, MD, USA

This is a very useful and important study about how to do fair and effective community engagement for field trials of genetically modified organisms.  

 The information obtained from this workshop will be useful to others who are planning community engagement activities. The article is well-written and well-researched.  

 I have no suggestions for improvement and look forward to its indexing.

Does the article adequately reference differing views and opinions?

Yes

Are all factual statements correct, and are statements and arguments made adequately supported by citations?

Yes

Is the Open Letter written in accessible language?

Yes

Where applicable, are recommendations and next steps explained clearly for others to follow?

Yes

Is the rationale for the Open Letter provided in sufficient detail?

Yes

Reviewer Expertise:

bioethics, research ethics, environmental ethics

I confirm that I have read this submission and believe that I have an appropriate level of expertise to confirm that it is of an acceptable scientific standard.
